# Phase angle is inversely related to the consumption of ultra-processed foods and positively related to the consumption of minimally processed foods by university students: a cross-sectional study

**DOI:** 10.1017/S136898002400123X

**Published:** 2024-09-13

**Authors:** Paraskevi Detopoulou, Despoina Levidi, Olga Magni, Vassilios Dedes, Milia Tzoutzou, Evaggelia Fappa, Aristea Gioxari, Georgios Panoutsopoulos

**Affiliations:** 1 Department of Clinical Nutrition, General Hospital Korgialenio Benakio, Athens, Greece; 2 Department of Nutritional Sciences and Dietetics, University of the Peloponnese, Kalamata 24100, Greece

**Keywords:** Phase angle, Ultra-processed foods, Minimally processed foods, Students

## Abstract

**Objective:**

Ultra-processed foods (UPF) and minimally processed foods (MPF) consumption are differentially connected to adiposity and possibly body composition. Phase angle (PhA) originates from bioelectrical impedance analysis (BIA) and is connected to cellular health. This study is the first to investigate associations between UPF/MPF consumption and PhA.

**Design:**

A cross-sectional study was conducted. Anthropometrical and BIA were performed. The Hellenic Physical Activity Questionnaire was used for physical activity evaluation, while a validated FFQ was used for dietary assessment. UPF and MPF intake (% energy) were determined according to the NOVA system. Partial correlation coefficients of PhA and dietary variables were assessed after multi-adjustment.

**Participants:**

Students were recruited (*n* 151, 114 women).

**Setting:**

University

**Results:**

Median and interquartile range (IQR) of PhA were 5·5° (5·1–6·4°) in the total sample, 6·8° (6·1–7·3°) in men and 5·3° (5·1–5·9°) in women (*P* < 0·001). The median and IQR for UPF consumption was 13·7 (8·1–33·4) % in the total sample, 23·8 (8·1–70·5) % in men and 12·9 (8·1–27·5) % in women (*P* < 0·001). The mean (sd) of MPF consumption was 60·2 (sd 15·7) % for the total sample, 59·1 (sd 16·4) % for men and 60·5 (sd 15·6) % for women (*P* = 0·720). The consumption of UPF was negatively (rho = –0·267, *P* = 0·002), while the consumption of MPF was positively (rho = 0·218, *P* = 0·010) associated with the PhA, after adjustment for age, sex, BMI and physical activity.

**Conclusion:**

PhA relates inversely to UPF and positively to MPF consumption. The observed associations possibly reflect the effects of diet on cellular health and in turn PhA.

The transition to university life may be associated with the purchase of ready-to-eat meals and the consumption of ultra-processed foods (UPF)^(1)^, which are usually highly palatable, energy-dense, unbalanced choices. UPF consumption has been inversely related to adherence to cardioprotective dietary patterns, such as the Mediterranean diet^(1)^ and an ‘early eating’ pattern^(1)^. In parallel, diets rich in UPF are high in sugars, salt, saturated and trans-fatty acids and low in fibre and potassium^(2)^. On the contrary, minimally processed foods (MPF) have a higher satiating ability and result in lower postprandial glycaemic peaks than UPF^(3)^. It is noteworthy that diets rich in UPF have been specifically related to central and visceral fat accumulation^(1,4)^. However, little data exist regarding their further association with other body composition parameters, such as lower muscle mass^(5)^.

Bioelectrical impedance analysis (BIA) constitutes a simple, non-invasive, low-cost method of body composition assessment^(6)^. Phase angle (PhA) derives from BIA and relates to cellular health with higher values reflecting better membrane integrity and cell function^(7)^. Its application as a prognostic index is increasing in several diseases such as CVD, cancer and others^(7,8)^. In a meta-analysis of 22 studies, subjects with CVD had lower values of PhA than healthy counterparts. Nutritional interventions in patients with cancer can also affect PhA along with muscle strength, according to a recent meta-analysis^(9)^. Indeed, PhA is a ‘sensitive’ index subjective to changes in cancer therapy^(10)^ and monitoring of muscle injury^(7)^. Moreover, its application in healthy individuals is broad, reflecting nutritional status, muscle status and body composition^(7)^. Moreover, PhA is considered an index of cell mass^(7)^, with higher values relating to higher body cell mass and cellular integrity^(7)^, while several factors also affect PhA, such as age, sex and fat-free mass^(11)^.

Given the utility of PhA as a nutritional index and a proxy of cellular health^(7)^, it is crucial to identify modifiable factors that can affect it. In this context, the potential role of dietary factors in PhA determination has been examined. In general, few studies have assessed the nutritional correlates of PhA. In a previous study of our group, a dietary pattern rich in potatoes, meat and poultry was positively related to PhA in patients with lung cancer^(12)^. Similarly, PhA has been positively associated with meat consumption in healthy subjects^(13)^, and it increases after a ketogenic diet irrespectively of weight loss^(14)^.

Interestingly, a higher adherence to anti-inflammatory dietary patterns, such as the Mediterranean diet, has been positively related to PhA^(15)^, while PhA values have been previously negatively related to inflammation^(16–18)^. Moreover, serum *n*-3 fatty acids, with known anti-inflammatory actions, have been positively correlated with PhA^(19,20)^. UPF consumption is inversely related to Mediterranean diet adherence^(1)^, and it increases inflammatory burden^(21)^ and oxidative stress^(22)^. Moreover, limited data have associated UPF consumption with lower muscle mass^(5,23)^.

Taking the effects of UPF and MPF at the cellular level as a starting point, it can be hypothesised that UPF and MPF consumption may differentially affect cellular health, body composition and PhA through modification of the oxidative and inflammatory milieu. Thus, the aim of the present study was to first investigate the relation of UPF and MPF consumption to PhA. The potential validation of such a hypothesis is important, since future clinical interventions aiming at maximising PhA could incorporate modifications in diet quality and changes in UPF and MPF consumption.

## Methods

### Study design and study sample

This is a cross-sectional study of university students. The measurements were performed in June 2022 and January 2023. The participants of the present study were undergraduate students. The vast majority of them were enrolled at the Department of Nutrition and Dietetics (> 98 %). Participants were selected by convenience sampling. Notices were electronically dispatched to the students by the Department of Nutrition and Dietetics secretariat, while advertisements were prominently displayed in frequented University areas, like the cafeteria and the restaurant zone. The eligibility criteria were (i) being a student at the University. Exclusion criteria were (i) not being a student, (ii) not fulfilling prerequisites for BIA measurement (i.e. no strenuous exercise, caffeine and alcohol intake for 8 h before measurement, no foods, liquids for 3 h before measurements, measurements not performed during menses and +/–3 d to avoid oedemas) and (iii) pregnancy. It is noted that no specific age inclusion/exclusion criteria have been applied. The STROBE-nut reporting checklist is given as an online supplementary material, Supplemental File.

### Evaluation of dietary habits and diet quality

A semi-quantitative FFQ was administered^(24)^. The FFQ covered a period of one year and consisted of 69 questions, concerning the frequency of intake of several food groups: dairy products, eggs, starchy vegetables, meat and poultry, fish, legumes, vegetables, fruits, sweets, alcohol, stimulants, fats and oils^(24)^. Most fruits and vegetables included in the FFQ were available throughout the whole year. To capture the seasonal consumption of fruits, two special questions were present, one for each season (summer fruits and winter fruits). The questionnaire, also, contained 7 questions regarding dietary habits, such as the use of oils and butter in cooking, the intake of visible fat from meat, the frequency of ordering food, the frequency of meals, the total number of meals including snacks, the number of main meals, the preference for organic or soy products and the intake of nutritional supplements^(24)^. The possible frequencies, in multiple-choice format, were as follows: ‘never/rarely’, ‘one to three times a month’, ‘one to two times a week’, ‘three to six times a week’, ‘once a day’ and ‘more than twice a day’. For other questions on dietary habits or supplements, a ‘yes’ or ‘no’ format was used^(24)^. The FFQ has been validated in the participant population^(24)^.

One serving of cereals (refined or non-refined) was considered as 1 slice of bread, ½ cup rice or ½ cup pasta. One serving of potatoes was considered as 1 medium potato. One serving of fruit was considered 1 medium fruit or ¼ of a cup of dried fruits. One serving of vegetables was considered as 1 cup of raw vegetables, ½ cup of cooked vegetables, ½ plate of spinach rice/vegetable rice or 2 slices of vegetable pie (e.g. spinach pie). One serving of legumes was considered 1/2 plate of legumes. One serving of fish was considered 60 g of fish. One serving of red meat was considered as 60 g beef/pork lamb/goat, 2 slices of ham, 2 medium sausages or 4 slices of bacon. One serving of poultry was considered as 60 g of chicken or turkey. One serving of eggs was considered 1 egg. One serving of dairy (full-fat or low-fat) was considered as 1 glass of milk or 1 yogurt or 30 g of cheese/cream cheese. One serving of sweets/treats was considered as 1 piece of pastry/tart, 1 whole croissant, 1 medium wafer, 1 slice of cake, 3–4 cookies, 1 ice cream or 1 milkshake. One serving of potato chips/popcorn was considered as 70 g of potato chips/popcorn. One serving of alcohol was considered as 125 ml of wine (1 glass), 240 ml of beer (1 glass) or 1 glass of other alcoholic drinks (30 ml).

Participants’ responses to the FFQ were also used to calculate the Mediterranean Diet Score (MedDietScore)^(25)^. MedDietScore evaluates the individual’s adherence to the Mediterranean Diet, and it is based on the frequency of consumption of the main food groups, on a scale from 0 to 55^(25)^.

In addition, the administered FFQ was used to calculate the percentage of energy derived from MPF and UPF, as defined by the NOVA system^(26)^. Based on the NOVA classification system, foods are divided into the following 4 categories according to their degree of processing: MPF or non-processed foods, processed cooking ingredients, processed foods and, finally, UPF^(26)^. MPF or non-processed foods are eaten ‘as they are’ or have undergone negligible industrial processing, such as fresh or dried or frozen fruits and vegetables, fresh, chilled or frozen meat, fish, eggs and dairy products. They are categorised as class ‘1’ foods. UPF, rated as class ‘4’, are those that have undergone a series of industrial processes, such as carbonated soft drinks, most breakfast cereals and pastries^(26)^. Then, the energy contribution of each category was calculated (as % of total energy intake). The USDA database was used to determine the energy values of foods^(27)^.

### Assessment of physical activity

Participants were administered the Hellenic Physical Activity Questionnaire^(28)^. It is a self-completed one-page questionnaire that includes data about the physical activity of the previous seven days and has been adapted to the habits of the particular population^(28)^. The questions are divided into three sub-categories: physical activity at work, physical activity at home and physical activity for leisure, to increase the examinee’s recall^(28)^. Also, for a better assessment of total energy expenditure, habits related to sleep and sedentary life are recorded^(28)^. For each activity, the examinee recorded the amount of time spent on it per day^(28)^. Metabolic equivalents for each of the physical activities were calculated by the examiner^(28)^. The metabolic equivalents were added, and finally, the individual’s energy expenditure was calculated, which was expressed as kilocalories per week and kilocalories per day^(28)^.

### Anthropometry and body composition

Body weight was measured with a precision scale (Tanita MC-780, Japan) to the nearest 0·1 kg, and height was measured to the nearest 0·1 cm with a stadiometer (Seca, Hamburg, Germany). The BMI was calculated as weight (in kilograms) divided by height squared (in metres squared) and the students were categorised as underweight, normal weight, or overweight/obese according to WHO criteria. More specifically, underweight students were those with BMI < 18·5 kg/m^2^, normal weight were those with BMI of 18·5–24·99 kg/m^2^, overweight were those with BMI of 25–29·99 kg/m^2^, and obese were those with BMI > 30 kg/m^2^. Waist and hip circumferences were measured with a non-stretchable measuring tape (Seca, Hamburg, Germany) to the nearest 0·1 cm, in a standing position^(29)^. When measuring the waist circumference, the measuring tape was placed around the narrowest part of the waist, that is between the last rib and above the level of the navel, while, when measuring the hip circumference, it was placed at a level that ensures that maximum hip circumference is measured, which is at the buttock area.

Body composition and PhA were assessed via multi-frequency BIA (Tanita MC-780, Japan). The device used for BIA measurements is a multi-frequency segmental body composition model, measuring at 5 kHz, 50 kHz and 250 kHz. In the present work, we have used PhA automatically calculated by the device at 50 kHz for all participants, while resistance (R) and reactance (Xc) were also provided. The day before BIA measurements subjects refrained from strenuous exercise (for 8 h), and they did not consume beverages high in caffeine (tea, coffee and energy drinks) nor alcohol^(29)^. On the day of measurements, subjects had fasted for at least 3 h (no foods and no liquids)^(29)^. Measurements were not performed during menses and +/–3 d to avoid water retention, which could potentially affect measurements. In order to perform measurements, shoes and socks were removed^(29)^. Measurements were performed in a standing position. More particularly, subjects stepped on the device with the feet touching the electrodes and the weight of clothes was recorded and automatically subtracted from the total weight measured by the device. When indicated, the subject put his hands on the electrodes, slightly separated from the trunk^(29)^, and the measurement was taken.

Lastly, skinfolds were measured at various anatomical sites (biceps, triceps, subscapular and supra-iliac skinfolds) according to standard procedures^(29)^. The same caliper was used for all measurements (Slim Guide Caliper, HaB Essentials). The caliper was operated with the right hand, and measurements were made on the right side of the subject’s body. The examiner with the thumb and forefinger of his left hand had to grasp approximately 1 cm above and below the measurement point at the respective anatomical site and separated the skin from the underlying muscle tissue^(29)^. After four seconds of applying pressure, the reading was recorded to the nearest 0·2 mm^(29)^. The measurement was performed 3 times at each point to minimise measurement error, and the mean value was used. For the measurement of biceps and triceps skinfolds, the midpoint of the arm length (between the acromion and olecranon) was identified and marked^(29)^. Then, vertical folds were taken at mid-biceps and mid-triceps levels^(29)^. The subscapular skinfold measurement was diagonally taken at 1 cm below the inferior angle of the scapula^(29)^, after locating the anatomical site by palpation of the area. For the supra-iliac skinfold, a diagonal fold, just above the front forward protrusion of the hip bone, was considered^(29)^.

All measurements were performed by two members of the technical staff of the Department of Nutrition and Dietetics.

### Statistical analysis

For normality testing, the Kolmogorov–Smirnoff test was used. Normally distributed variables are shown as means (sd), and non-normally distributed variables are shown as medians and interquartile range. Categorical variables are presented as frequencies (*n*, %). For comparisons between men and women, the *t* test was applied for normally distributed variables or the Mann–Whitney *U* test for non-normally distributed variables. For categorical variables, the chi-square test was used to compare frequencies between sexes.

Correlations between variables were performed using the Spearman correlation coefficient, since in most variables the normality criterion was not fulfilled. For the calculation of Spearman’s partial correlation coefficients, variables were ranked, and then, the Pearson partial correlation coefficients were calculated, after adjustment for age, sex, physical activity and BMI. Furthermore, Spearman’s partial correlation coefficients were reported in several groups, that is MedDietScore tertiles (low, medium and high adherence to the Mediterranean diet).

All reported *P*-values are based on two-sided tests, and the level of significance was set at 5 %. IBM SPSS Statistics for Windows 29.0.0.0.241(IBM Corp.) was used for analysis.

## Results

### Basic characteristics of the participants

In total, 151 students participated, including 114 women and 37 men, aged between 18 and 44. The baseline characteristics of the participants are presented in Table 1. It is noted that overweight and obese subjects were grouped, due to the low frequency of obese subjects. Women had a higher percentage of body fat and higher thickness levels of several skinfolds. Extracellular and intracellular water, as well as lean mass, were higher in male participants. Waist circumference was higher in men. The PhA was 6·8 (6·15–7·35) in men and 5·3 (5·1–5·9) in women (medians, interquartile ranges *P* < 0·001).

**Table 1:** Descriptive characteristics of participants

	Total			Men			Women			
	*n*	Mean	sd	*n*	Mean	sd	*n*	Mean	sd	*P*-value
Number of participants (n)	151			37			114			
Age (years)		20·88	4·1		20·89	4·35		20·88	4·04	0·783
Year of studies										
1st (*n*)	45			14			31			0·3
2nd (*n*)	47			15			32			0·2
3rd (*n*)	44			4			40			0·004
4th (*n*)	12			4			8			0·5
		*n*	%		*n*	%		*n*	%	
Current smoking (*n*, %)		30	19·8		7	18·9		23	20·1	0·538
Non-smokers (*n*, %)		117	77·4		28	75·6		89	78·0	0·822
Former smokers (*n*, %)		4	2·6		2	5·4		2	1·7	0·252
		Median	IQR		Median	IQR		Median	IQR	
Years of smoking		4	2·75–6·25		6	4–6		3	2–5	0·124
Cigarettes per day (number)		5	7–12·5		10	7–10		5·5	4–10·2	0·152
		Mean	sd		Mean	sd		Mean	sd	
BMI (kg/m^2^)		22·0	2·9		22·8	3·1		21·7	2·8	0·040
Underweight (*n*)	15			2			13			0·3
Normal weight (*n*)	115			26			89			0·3
Overweight and Obese (*n*)	21			9			12			0·03
		Median	IQR		Median	IQR		Median	IQR	
Lean body mass (kg)		44·7	40·6–51·6		63·2	55·5–67·1		42·4	40·2–46·2	<0·001
		Mean	sd		Mean	sd		Mean	sd	
Fat mass (%)		23·0	7·6		15·65	7·05		25·51	6·09	<0·001
Fat mass (kg)		14·2	5·6		11·5	5·4		15·2	5·4	<0·001
		Median	IQR		Median	IQR		Median	IQR	
Intracellular water (kg)		17·3	15·9–19·5		25·9	23·4–28·2		16·7	15·6–17·7	0·024
Extracellular water (kg)		13·2	12·2–15·6		17·4	16·2–18		12·7	12·1–13·6	<0·001
PhA (°)		5·5	5·1–6·4		6·8	6·1 – 7·3		5·3	5·1 – 5·9	<0·001
Wrist Circumference (cm)		15·5	14·6–16·4		17·0	16·3–18·0		15·0	14·5–16·0	<0·001
Waist Circumference (cm)		71·0	67·0–75·0		78·0	74·0–81·0		69·0	66·0–72·0	<0·001
Hip Circumference (cm)		97·0	93·0–101·0		98·0	95·0–103·0		97·0	92·0–101·0	0·057
Biceps skinfold (mm)		8·5	5·3–12·5		5·0	4·0–7·0		9·25	6·6–13·1	<0·001
Triceps skinfold (mm)		14·9	12·0–20·0		11·0	8·8–15·1		15·6	13·3–20·3	<0·001
Subscapular skinfold (mm)		11·4	9·6–15·3		11·5	9·3–14·65		11·3	9·6–15·3	0·762
Supra-iliac skinfold (mm)		10·5	8·0–13·9		9·6	5·9–12·3		11·0	8·3–14·0	0·031
Physical activity										
Total MET/min per day		1832	1689–2034		1844	1741·5–2196·5		1821·5	1682·5–1996·5	0·277
Sleep duration (hours)		7·0	6·0–8·0		7·5	6·2–8·0		7·0	6·0–8·0	0·899
Screen time										
Time watching TV/video (hours)		3·0	2·0–4·0		2·0	2·0–3·5		3·0	2·0–4·0	0·288
Time on the computer (hours)		5·0	2·0–12·0		5·0	3·0–12·0		5·5	2·0–10·5	0·727

IQR: interquartile range; MET: Metabolic equivalents; PhA: Phase angle.

Data are presented as means (sd) for normally distributed variables or as median and interquartile range, for non-normally distributed variables. Categorical variables are displayed as *n*. *t* test (for normal variables) or Mann–Whitney *U* test (for non-normal variables) was used to compare values between men and women. The chi-square test (for categorical variables) was used to compare categorical variables between men and women.

### Dietary intake of the participants

The dietary intake of the participants is shown in Table 2. The medians and interquartile ranges of the MedDietScore, UPF and MPF intake were 31·00 (26·00–34·00), 13·72 (8·18–33·39) and 59·99 (50·16–70·73), respectively, in the total sample. No sex differences were documented in these parameters, while men had higher red meat intake than women. The correlation coefficients of UPF and MPF with food groups are shown in online supplementary material, Supplemental Tables 1 and 2.

**Table 2:** Consumption of food groups and eating habits

	Total Sample (*n* 151)		Men (*n* 37)		Women (*n* 114)		
Parameters evaluated	Median	IQR	Median	IQR	Median	IQR	*P*-value
Refined cereals (servings per day)	2·18	1·41–3·48	1·62	1·26–3·48	2·18	1·55–3·50	0·267
Non-refined cereals (servings per day)	0·64	0·19–1·42	0·62	0·09–1·42	0·64	0·19–1·44	0·369
Potatoes (servings per day)	0·26	0·11–0·36	0·26	0·18–0·68	0·21	0·11–0·36	0·108
Fruits (servings per day)	1·77	0·83–3·20	1·71	0·94–3·09	1·77	0·77–3·22	0·943
Vegetables (servings per day)	1·95	0·95–2·77	1·47	0·75–2·47	2·01	1·02–2·79	0·055
Legumes (servings per day)	0·53	0·16–0·53	0·53	0·16–0·53	0·53	0·16–0·53	0·388
Fish (servings per day)	0·33	0·16–0·53	0·33	0·00–0·53	0·33	0·16–0·53	0·577
Red meat (servings per day)	0·99	0·56–1·80	1·61	0·91–2·34	0·90	0·49–1·65	0·002
Poultry (servings per day)	0·53	0·53–1·60	0·53	0·53–1·60	0·53	0·53–1·60	0·764
Eggs (servings per day)	0·21	0·21–0·64	0·21	0·21–0·64	0·42	0·21–0·64	0·925
Full-fat dairy (servings per day)	0·64	0·21–1·00	0·64	0·13–1·14	0·64	0·21–0·85	0·540
Low-fat dairy (servings per day)	0·28	0·06–1·00	0·21	0·06–0·85	0·53	0·06–1·00	0·388
Sweets/Treats (servings per day)	0·56	0·26–0·98	0·47	0·27–0·84	0·56	0·26–1·05	0·571
Chips/Popcorn (servings per day)	0·06	0·00–0·21	0·06	0·00–0·14	0·06	0·00–0·21	0·737
Alcohol (servings per day)	0·21	0·06–0·49	0·19	0·06–0·49	0·24	0·06–0·49	0·896
Energy intake (kcal per day)	1920	1647–2179	2229	1960–3054	1827	1603–2078	<0·001
MedDietScore	31·0	26·0–34·0	29·0	26·0–34·0	31·0	27·0–34·00	0·172
UPF (% of energy per day)	13·72	8·18–33·39	23·84	8·18–70·51	12·93	8·11–27·54	0·075
	Mean	sd	Mean	sd	Mean	sd	
MPF (% of energy per day)	60·21	15·76	59·12	16·41	60·58	15·60	0·629
	*n*	%	*n*	%	*n*	%	
Meals per day							0·004
1–3	43	28·5	10	27	33	28·9	
4–5	101	67	22	59·5	79	69·3	
6 or more	6	4	5	13·5	1	0·9	
Main meals per day (breakfast, lunch and dinner)							0·137
One main meal	11	7·3	5	13·5	6	5·3	
Two main meals	60	39·7	11	29·7	49	43	
Three main meals	76	50·3	20	54·1	56	49·1	
Eating breakfast							0·721
Never/Rarely	8	5·3	3	8·1	5	4·4	
1–3 times per month	11	7·3	2	5·4	9	7·9	
1–2 times per week	15	9·9	3	8·1	12	10·5	
3–6 times per week	29	19·2	9	24·3	20	17·5	
Once per day	86	57	19	51·4	67	58·8	
Consumption of organic or soy products							
Yes	59	39·1	10	27	49	43	
No	90	59·6	26	70·3	64	56·1	
Taking nutritional supplements (e.g. vitamins)							
Yes	55	36·4	16	43·2	39	34·2	
No	95	62·9	21	56·8	74	64·9	

IQR: interquartile range; MPF: minimally processed foods; UPF ultra-processed foods.

Data are presented as medians and interquartile ranges, for non-normally distributed variables. Categorical variables are displayed as *n* (%). Mann–Whitney *U* test (for non-normal variables) was used to compare values between men and women. The chi-square test (for categorical variables) was used to compare categorical variables between men and women.

### Relation of phase angle with dietary variables

**Fig. 1:**
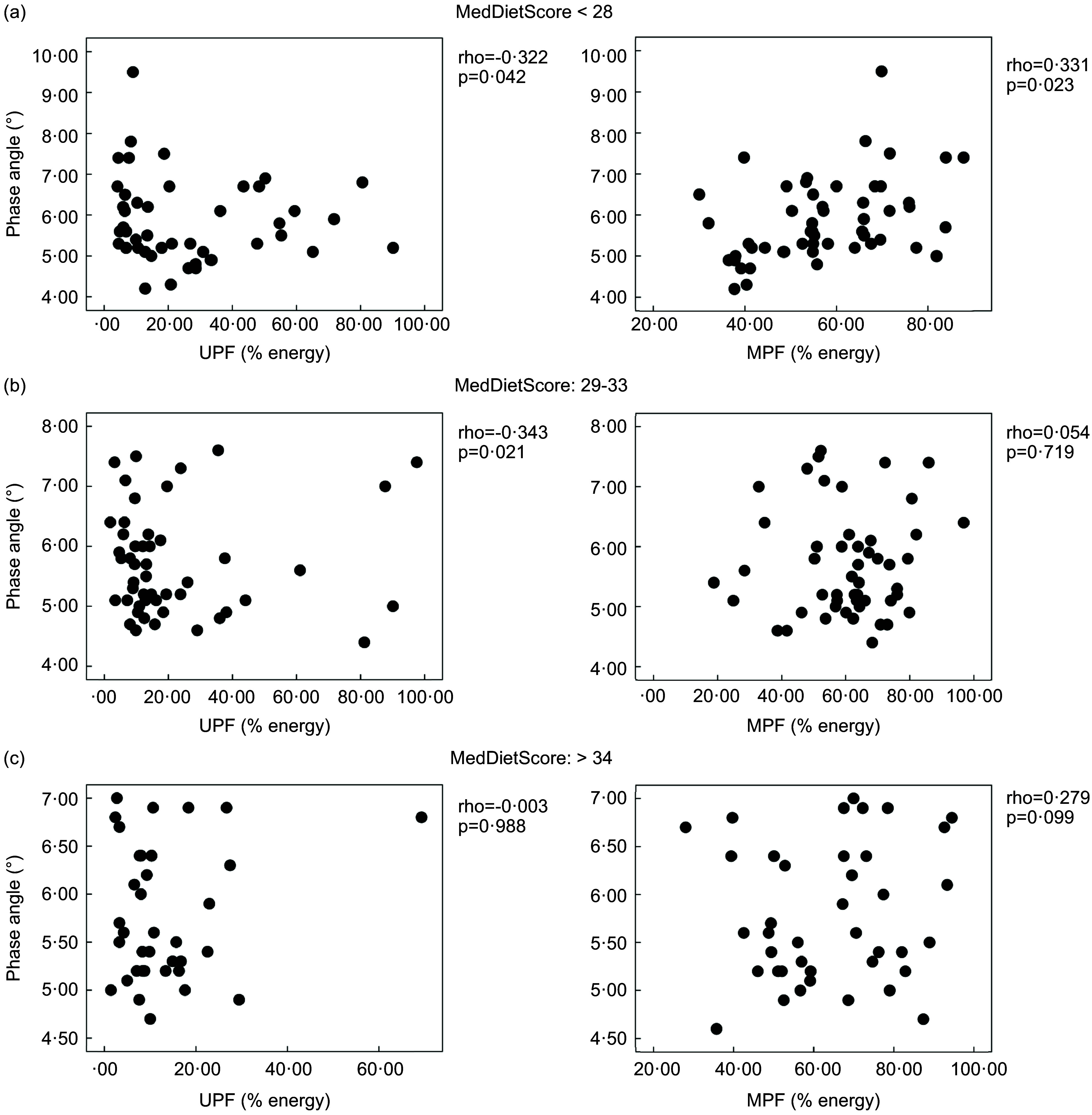
Pearson’s partial correlations between PhA and dietary variables (ranked variables) after adjustment for age, physical activity, BMI and sex stratified by MedDietScore tertile

In Table 3, the Spearman correlations between PhA and dietary variables are presented. The association of UPF with PhA was significant in women, while the association of MPF with PhA was significant in both sexes. In a multivariate analysis using Pearson’s partial correlations between PhA and dietary variables (ranked variables) after adjustment for age, physical activity, BMI and sex (where applicable), the associations remained significant (Table 4). It is noted that the associations also remained significant in further models adjusted for muscle mass instead of BMI (rho = –0·253, *P* = 0·004 and rho = 0·228, *P* = 0·007 for the association of PhA with UPF and MPF, correspondingly). The associations were further tested in stratified analysis per MedDietScore tertile (Fig. 1). It was shown that the inverse association of UPF and PhA was evident in participants who had low and moderate adherence to the Mediterranean diet (i.e. first and second tertile of the MedDietScore). Interestingly, the positive association of MPF consumption and PhA was evident in those with low adherence to the Mediterranean diet (first tertile of the MedDietScore).

**Table 3:** Spearman’s correlations between PhA and dietary variables

	Total sample (*n* 151)		Men (*n* 37)		Women (*n* 114)	
	Correlation coefficient	*P*-value	Correlation coefficient	*P*-value	Correlation coefficient	*P*-value
Refined cereals (servings per day)	−0·062	0·452	0·043	0·799	0·029	0·766
Non-refined cereals (servings per day)	−0·042	0·615	−0·171	0·311	0·073	0·449
Potatoes (servings per day)	0·043	0·607	−0·049	0·775	−0·068	0·480
Fruit (servings per day)	0·090	0·276	0·266	0·111	0·060	0·529
Vegetables (servings per day)	−0·054	0·515	0·005	0·978	0·020	0·837
Legumes (servings per day)	−0·050	0·550	0·070	0·680	−0·058	0·548
Fish (servings per day)	0·074	0·373	0·259	0·121	−0·030	0·756
Red meat (servings per day)	0·173	0·035	0·039	0·818	0·017	0·861
Poultry (servings per day)	0·023	0·784	0·037	0·830	0·007	0·943
Eggs (servings per day)	−0·032	0·700	0·081	0·632	−0·036	0·707
Full-fat dairy (servings per day)	0·039	0·638	0·080	0·640	−0·006	0·954
Low-fat dairy (servings per day)	−0·016	0·847	−0·061	0·722	0·092	0·338
Sweets/Treats (servings per day)	−0·043	0·602	0·061	0·719	−0·051	0·593
Chips/Popcorn (servings per day)	0·045	0·583	−0·067	0·693	0·107	0·262
Alcohol (servings per day)	0·066	0·425	0·082	0·631	0·069	0·471
MedDietScore (0–55)	−0·048	0·569	0·097	0·567	−0·018	0·854
UPF (% of Energy per day)	−0·166	0·058	−0·116	0·527	−0·343	0·001
MPF (% of Energy per day)	0·189	0·025	0·364	0·027	0·239	0·014

MedDietScore: Mediterranean diet score; MPF: minimally processed foods; UPF ultra-processed foods.

Spearman’s correlation coefficients between phase angle and dietary parameters.

**Table 4:** Pearson’s partial correlations between PhA and dietary variables (ranked variables) after adjustment for age, physical activity, BMI and sex (where applicable)

		Total	Men (*n* 37)		Women (*n* 114)	
	Correlation coefficient	*P*-value	Correlation coefficient	*P*-value	Correlation coefficient	*P*-value
UPF (% of Energy per day)	−0·277	0·002	−0·112	0·562	−0·339	0·001
MPF (% of Energy per day)	0·218	0·010	0·329	0·058	0·213	0·032

MPF: minimally processed foods; UPF ultra-processed foods.

Spearman’s correlation coefficients between phase angle and dietary parameters.

## Discussion

The present study firstly documented that the consumption of UPF was negatively related to PhA, while the consumption of MPF was positively associated with PhA in a sample of university students. The associations were more prominent in participants with low-to-moderate adherence to the Mediterranean diet.

The UPF consumption in the present study (13·72 %) was similar to that reported in Italy (10 %)^(30)^, lower than that reported for Spain^(31)^ and France^(32)^ and much lower than that reported for the UK (more than 55%)^(33)^. Moreover, the intake of UPF was lower than that reported in a previous study of our group of university students conducted in 2018 (mean UPF intake 40·7 %)^(1)^. The median MPF intake was 59·9 % in the present and 44·3 % (mean) in our previous study^(1)^. These differences can be explained by (i) the fact that participants were mainly dietetic students, while in the previous study students from other disciplines participated (dietetic students may have better dietary habits or may selectively misreport unhealthy UPF foods)^(1)^ and (ii) the time period that the present study was conducted (in the COVID-19 epidemic). A recent analysis showed that UPF provided 25·2% of energy in a typical Greek hospital menu^(34)^.

Regarding PhA, it is noted that most subjects had low PhA compared to reference values^(35)^. This finding is probably attributed to the measurement procedure since lower PhA values are documented in standing position compared to lying position (with the same device)^(36)^. It is also possible that particularities exist in the present sample and that population-specific reference values would be more appropriate to compare with, as suggested in other studies^(37)^. In addition, device-specific reference values have been also proposed^(38)^.

As far as the observed associations between UPF, MPF and PhA are concerned, there are limited data in the literature. The vast majority of available data have focused on the relation of UPF with obesity and/or waist circumference^(1,4)^. To our knowledge, there is only one study assessing the relationship between UPF/MPF and PhA in 24 patients with inflammatory bowel disease and 21 controls^(39)^. This study showed non-significant correlations between PhA and the degree of food processing, possibly because of the small sample size and/ or clinical condition^(39)^. In a previous study of our group, a dietary pattern rich in potatoes, meat and poultry was positively related to PhA in patients with lung cancer^(12)^. Similarly, PhA has been positively associated with meat consumption in healthy subjects^(13)^ but not all studies agree^(40)^. The association of meat and total protein intake may be related to PhA as it is directly related to muscle mass^(41)^. In turn, PhA is positively associated with muscle mass in all age groups^(42)^. In this context, the association of meat with PhA (although red meat is not considered a healthy food choice) is possibly explained through the relation of meat intake with muscle mass. It is also noteworthy that the correlation of PhA with meat was evident only in the whole sample and not in sex-specific analysis. This implies that body composition variables may be responsible for the correlation of meat and PhA in the whole sample (i.e. men who have a higher meat intake and a higher muscle mass have higher values of PhA, and thus, a correlation is driven in the whole sample).

In parallel, a higher adherence to the Mediterranean diet (rich in MPF, such as fruits, vegetables and legumes) along with a diet with high antioxidant capacity has been positively related to PhA^(15,43)^. In addition, serum long-chain *n*-3 fatty acids (found mainly in fish) have been positively correlated with PhA^(19,20)^. Other studies regarding the association of UPF with body composition parameters have shown that the intake of UPF correlates with visceral fat (but not total fat) in women^(44)^ and lower muscle mass in young subjects^(5)^. It is noted, however, that the observed associations were independent of muscle mass since this variable was entered in multivariate models.

To better interpret this study’s results, the observed relation of UPF and MPF to PhA should be considered through the prism of food effects on oxidative stress, inflammation and cellular health. Indeed, the intake of UPF has been associated with increased oxidative stress^(22)^ and inflammatory burden^(21)^. Moreover, trans-fats and simple carbohydrates found in UPF have been documented to promote the translocation of NF-κB and activator protein-1^(45)^ and relate to circulating C-reactive protein, IL-6 and TNF-α^(46)^. Similarly, *n*-6 fatty acids, often found in processed foods in the form of sunflower or other oils, have been positively related to biosynthetic enzymes of the platelet-activating factor, which is a strong mediator of inflammation^(47)^. On the contrary, a diet rich in antioxidants and in unprocessed foods (i.e. fruits, nuts, herbal drinks, olive oil and whole-wheat products) has been related to reduced inflammatory markers^(48)^.

In parallel, PhA is positively related to antioxidant status, as it is correlated with glutathione concentration and superoxide dismutase activity^(39)^, while it is also negatively related to inflammatory markers, including platelet-activating factor^(16–18,43)^. It can be thus hypothesised that UPF and MPF can differentially affect the antioxidant status and cellular inflammatory milieu, which in turn directly or indirectly affects cellular health and PhA.

Interestingly, we herein first report that the association of UPF and MPF with PhA may be influenced by background diet since the association was mostly present in subjects with low-to-moderate Mediterranean diet adherence. This implies that subjects following an unhealthy dietary pattern could benefit from the reduction of UPF consumption. The importance of background diet has been also shown in a previous study of our group^(49)^. Indeed, we have previously shown that the dietary antioxidant capacity related to glycaemic indices only in subjects being away from the Mediterranean diet^(49)^. However, these observations need further investigation.

The main strength of the present study pertains to the body composition analysis performed and PhA measurements, which were not available in previous studies of our group concerning UPF intake^(1)^. Moreover, the sample was quite homogenous, since mainly dietetics students participated.

However, several limitations should be reported. This work is a cross-sectional study. Therefore, causal relationships between the factors cannot be substantiated. In addition, the sample consisted exclusively of students of a single university. Most participants were students in the Department of Nutrition, which could impact their dietary choices and nutrition knowledge. Moreover, the present sample consisted mainly of women. All the aforementioned factors limit the generalisability of the present findings. Regarding the participants’ eating habits and diet quality, the assessment was based on the participants’ responses to the self-administered FFQ. Therefore, the results may be affected by the recall ability of the subjects and the possibility of under- or over-reporting cannot be excluded. Several factors influencing BIA measurements have been reported, which are related to the instrument, the technician performing the measurements, especially when patches are applied, the subject and the environmental temperature^(6)^. However, in the present study, the same machine was used. Eight contact electrodes were used, which provide reliable measurements compared to previous TANITA models^(50)^. On top of this, electrodes are not placed by a technician, which minimises the interference of technician-related errors. In addition, all manufacturers’ recommendations on measurement conditions regarding hydration, food intake, recent physical activity and menstrual cycle were followed^(6)^.

In conclusion, the correlations of PhA with the consumption of UPF (negative correlation) and MPF (positive correlation) were first reported in this study. These associations are possibly due to the interaction of nutrition with inflammation and oxidative stress mechanisms, which in turn affect cellular health and PhA. Future studies with a larger and more representative sample including subjects with a broader age range should be conducted, to affirm or refute our findings.

## Supporting information

Detopoulou et al. supplementary material Detopoulou et al. supplementary material

## Supplementary material

For supplementary material accompanying this paper, visit https://doi.org/10.1017/S136898002400123X
