# Supplementary material for: Phase angle is inversely related to the consumption of ultra-processed foods and positively related to the consumption of minimally processed foods by university students: a cross-sectional study
Source: Public Health Nutr. 2024 Sep 13;27(1):e161. doi: 10.1017/S136898002400123X (PMC11504085; doi:10.1017/S136898002400123X)
Supplement: Detopoulou et al. supplementary material [file S136898002400123Xsup001.docx]

| **Supplementary Table 1: Spearman correlations between UPF consumption and food groups.** | | | | | | |
| --- | --- | --- | --- | --- | --- | --- |
|  | **Total Sample**  (n=151) | | **Men**  (n=37) | | **Women**  (n=114) | |
|  | Correlation coefficient | p-value | Correlation coefficient | p-value | Correlation coefficient | p-value |
| Refined cereals (servings per day) | -0.041 | 0.637 | -0.166 | 0.363 | -0.003 | 0.973 |
| Non-refined cereals (servings per day) | -0.181 | 0.036 | -0.213 | 0.241 | -0.122 | 0.221 |
| Potatoes (servings per day) | 0.117 | 0.179 | 0.046 | 0.804 | 0.122 | 0.220 |
| Fruits (servings per day) | -0.133 | 0.126 | -0.102 | 0.579 | -0.115 | 0.249 |
| Vegetables (servings per day) | -0.214 | 0.013 | -0.274 | 0.129 | -0.163 | 0.102 |
| Legumes (servings per day) | -0.216 | 0.012 | -0.317 | 0.077 | -0.192 | 0.053 |
| Fish (servings per day) | -0.061 | 0.483 | -0.375 | 0.035 | 0.055 | 0.583 |
| Red meat (servings per day) | 0.283 | 0.001 | 0.193 | 0.291 | 0.304 | 0.002 |
| Poultry (servings per day) | 0.052 | 0.547 | -0.081 | 0.658 | 0.131 | 0.189 |
| Eggs (servings per day) | 0.006 | 0.947 | <0.001 | 0.998 | 0.016 | 0.869 |
| Full-fat dairy (servings per day) | 0.109 | 0.208 | -0.206 | 0.258 | 0.237 | 0.016 |
| Low-fat dairy (servings per day) | -0.082 | 0.348 | -0.162 | 0.376 | -0.070 | 0.486 |
| Sweets/Treats (servings per day) | 0.055 | 0.527 | 0.116 | 0.529 | 0.061 | 0.541 |
| Chips/Pop-corn (servings per day) | 0.051 | 0.556 | 0.158 | 0.386 | 0.026 | 0.794 |
| Alcohol (servings per day) | 0.121 | 0.164 | 0.282 | 0.118 | 0.096 | 0.339 |
| Spearman correlation coefficients between ultra-processed foods and food groups.  UPF: ultra-processed foods | | | | | | |

| **Supplementary Table 2: Spearman correlations between MPF consumption and food groups.** | | | | | | |
| --- | --- | --- | --- | --- | --- | --- |
|  | **Total Sample**  (n=151) | | **Men**  (n=37) | | **Women**  (n=114) | |
|  | Correlation coefficient | p-value | Correlation coefficient | p-value | Correlation coefficient | p-value |
| Refined cereals (servings per day) | 0.027 | 0.743 | -0.090 | 0.596 | 0.091 | 0.350 |
| Non-refined cereals (servings per day) | 0.049 | 0.555 | -0.068 | 0.691 | 0.079 | 0.417 |
| Potatoes (servings per day) | -0.263 | 0.001 | -0.141 | 0.404 | -0.285 | 0.003 |
| Fruits (servings per day) | 0.269 | 0.001 | 0.106 | 0.531 | 0.303 | 0.001 |
| Vegetables (servings per day) | 0.065 | 0.440 | -0.048 | 0.776 | 0.090 | 0.353 |
| Legumes (servings per day) | 0.055 | 0.513 | 0.005 | 0.975 | 0.063 | 0.518 |
| Fish (servings per day) | -0.060 | 0.475 | 0.100 | 0.556 | -0.119 | 0.220 |
| Red meat (servings per day) | -0.329 | <0.001 | -0.192 | 0.255 | -0.366 | <0.001 |
| Poultry (servings per day) | -0.022 | 0.792 | 0.222 | 0.187 | -0.112 | 0.248 |
| Eggs (servings per day) | 0.011 | 0.893 | -0.088 | 0.605 | 0.042 | 0.664 |
| Full-fat dairy (servings per day) | -0.087 | 0.300 | 0.187 | 0.266 | -0.192 | 0.047 |
| Low-fat dairy (servings per day) | 0.009 | 0.915 | 0.038 | 0.824 | 0.010 | 0.917 |
| Sweets/Treats (servings per day) | 0.019 | 0.817 | -0.095 | 0.578 | 0.059 | 0.546 |
| Chips/Pop-corn (servings per day) | 0.023 | 0.786 | -0.089 | 0.602 | 0.064 | 0.511 |
| Alcohol (servings per day) | -0.164 | 0.048 | -0.257 | 0.125 | -0.126 | 0.192 |
| Spearman correlation coefficients between minimally-processed foods and food groups.  MPF: minimally processed foods | | | | | | |
